# Health Information Technology: Meaningful Use and Next Steps to Improving Electronic Facilitation of Medication Adherence

**DOI:** 10.2196/medinform.4326

**Published:** 2016-03-15

**Authors:** Hayden B Bosworth, Leah L Zullig, Phil Mendys, Michael Ho, Troy Trygstad, Christopher Granger, Megan M Oakes, Bradi B Granger

**Affiliations:** ^1^ Duke University Medical Center Department of Medicine Durham, NC United States; ^2^ Center for Health Services Research in Primary Care, Durham VAMC Durham, NC United States; ^3^ Eshelman School of Pharmacy, University of North Carolina at Chapel Hill Chapel Hill, NC United States; ^4^ Division of Cardiology, Department of Medicine, University of Colorado Denver, CO United States; ^5^ VA Eastern Colorado Health Care System Denver, CO United States; ^6^ North Carolina Community Care Raleigh, NC United States; ^7^ Department of Medicine, Duke University Medical Center Durham, NC United States; ^8^ Duke Clinical Research Institute, Duke University Durham, NC United States; ^9^ School of Nursing and Health System, Duke University Durham, NC United States

**Keywords:** medication adherence, compliance, health information technology

## Abstract

**Background:**

The use of health information technology (HIT) may improve medication adherence, but challenges for implementation remain.

**Objective:**

The aim of this paper is to review the current state of HIT as it relates to medication adherence programs, acknowledge the potential barriers in light of current legislation, and provide recommendations to improve ongoing medication adherence strategies through the use of HIT.

**Methods:**

We describe four potential HIT barriers that may impact interoperability and subsequent medication adherence. Legislation in the United States has incentivized the use of HIT to facilitate and enhance medication adherence. The Health Information Technology for Economic and Clinical Health (HITECH) was recently adopted and establishes federal standards for the so-called "meaningful use" of certified electronic health record (EHR) technology that can directly impact medication adherence.

**Results:**

The four persistent HIT barriers to medication adherence include (1) underdevelopment of data reciprocity across clinical, community, and home settings, limiting the capture of data necessary for clinical care; (2) inconsistent data definitions and lack of harmonization of patient-focused data standards, making existing data difficult to use for patient-centered outcomes research; (3) inability to effectively use the national drug code information from the various electronic health record and claims datasets for adherence purposes; and (4) lack of data capture for medication management interventions, such as medication management therapy (MTM) in the EHR. Potential recommendations to address these issues are discussed.

**Conclusion:**

To make meaningful, high quality data accessible, and subsequently improve medication adherence, these challenges will need to be addressed to fully reach the potential of HIT in impacting one of our largest public health issues.

## Introduction

Non-adherence to prescription medications is common and costly [1]. Approximately 20-30% of prescription medications are never filled [1], with approximately 40% of patients failing to fill an initial prescription [2-4]. Even after a medication has been acquired, many patients do not follow prescription instructions. Within one year, over 50% of patients prematurely discontinue their medications [2,5,6]. The problem of medication non-adherence is complex and pervasive with a lack of accountability dispersed across patients, their caregivers, clinicians, pharmacy benefits, and the health care systems as a whole.

While there is no universal solution to improve medication adherence, health information technology (HIT) can inform and accelerate ongoing strategies to initiate, improve, and monitor medication adherence. Studies increasingly demonstrate that the use of HIT can improve the quality and coordination of care and lead to better health outcomes [7,8]. Two commonly cited examples of HIT are electronic health record (EHR) systems and electronic prescribing (e-prescribing), the electronic generation of a medication prescription and its routing to a pharmacy.

Recent legislation in the United States has incentivized the use of HIT to address medication adherence [9]. The Health Information Technology for Economic and Clinical Health (HITECH) Act was passed in 2009 as part of the American Recovery and Reinvestment Act. It authorized an estimated US $30 billion in incentives to eligible professionals and hospitals to adopt and meet federal standards for the so-called "meaningful use" of certified EHR technology [9]. Meaningful use in the context of medications includes EHR functions such as e-prescribing, creating linkages between patient diagnosis and treatment plan, generating reports for clinical quality measures, integrating clinical decision support, and providing electronic messaging between providers. Electronic patient portals also allow for secure messaging between patients and providers, potentially improving medication-related communication and care while reducing the frequency of traditional in-office visits. The Stage 2 core objectives include providing patients the ability to view online, download, and transmit their health information [10].

The Office of the National Coordinator recently proposed a version of the third stage of meaningful use, with a focus on both functionality and health care outcomes [11]. The Centers for Medicare and Medicaid Services (CMS) Stage 3 Meaningful Use proposed rule focuses on the use of EHR technology to promote improved patient outcomes and health information exchange. The rule proposes to ensure health systems and providers are coordinating care for patients, providing patients with easy access to their health information, and fostering data collection in a format that can be shared across multiple health care organizations. Early drafts of the Stage 3 Meaningful Use rule included medication adherence as well as several other related factors, such as medication reminders for refills. However, in early 2014, these were removed from the Stage 3 plan and are now included at the discretion of providers [12].

**Figure 1:**
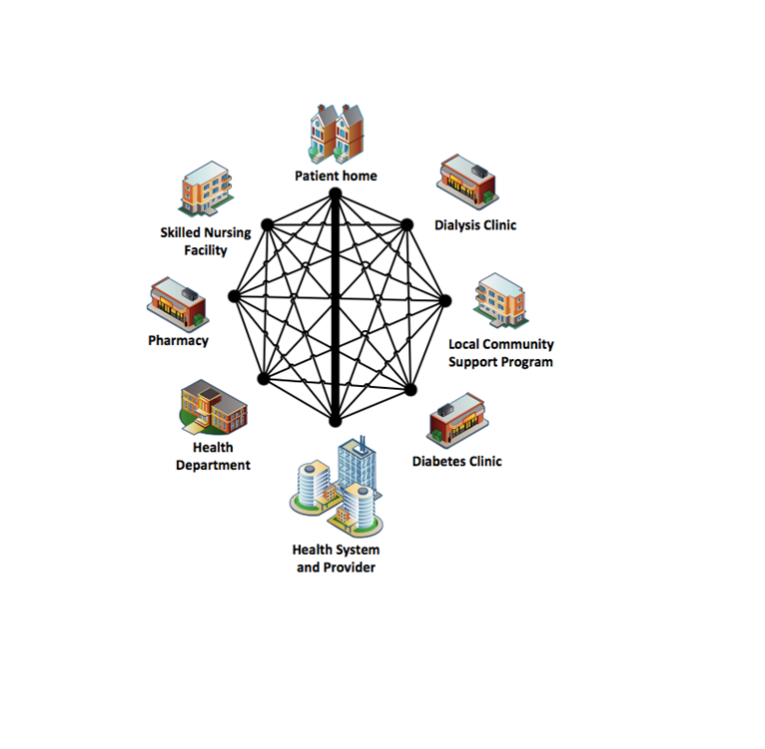
Gaps in electronic health record connectivity.

Meaningful Use Stage 3 would address key gaps identified in EHR functionality that impede improvement in clinical outcome and are considered essential for adoption by all providers (Figure 1). These key gaps include information exchange among provider entities, connectivity for patient engagement, and technology advances to reduce disparities by providing decision support for national high-priority conditions. Each of these gaps has implications for medication use, including opportunity to improve monitoring, documentation, communication, and feedback [12]. Thus, these proposed changes for Stage 3 Meaningful Use should streamline previous meaningful use criterion while improving health care quality.

Despite the well-intentioned design of certification criteria and clinical quality metrics, the status of medication adherence in the meaningful use guidelines is unclear. The purpose of this paper is to review the current state of HIT as it relates to medication adherence programs, acknowledge the potential barriers in light of current US legislation, and provide recommendations to improve ongoing medication adherence strategies through the use of HIT.

## Results

### Challenges of HIT Interoperability, Connectedness, and Reciprocity

Interoperability, defined as the extent to which systems and devices can exchange and interpret shared data [13], has been a longstanding challenge for meaningful use initiatives related to medication adherence. Lack of data connectivity and reciprocity (ie, the extent to which systems can interpret shared data, aka data exchange) across settings of care delivery has been identified as a key factor in poor data capture [14,15]. Specifically, interoperability that enables patients to share health information with their provider and/or health care system (eg, smart pill storage containers or blister packages that record and transmit when a medication has been taken and communicates with an EHR) in an actionable form is underdeveloped. As a result, patients are not able to "connect" data from self-monitoring efforts to their health records and providers are not able to evaluate these data in the context of the person’s other health information. In the case of medication adherence, there are a number of "users" in addition to the patient and provider. These include pharmacies, health systems, and payers. This network of "users" may become extensive; for example, patients may "price shop" and use multiple pharmacies to fill their prescriptions, intensifying the need to share information. As these stakeholders seek to evaluate and improve medication packaging and delivery devices the number of users in the network expands. In addition, their respective motives, goals, and willingness to participate in a transparent and interoperable data platform becomes less predictable. The end-result of a successful, "interoperable exchange" must be representation of data in a user-accessible format that allows users access to these data.

The problem of poor medication adherence HIT data reciprocity across systems takes the following two forms: (1) the complexity of the data, coding syntax, and the transmission infrastructure on which these data reside, and (2) the output, such as pill-taking history, medication refill rates or patient-reported experience of side effects. In both cases, these data are not accessible, timely, nor easily understood by the health care system. Thus, providers, patients, family members, and community support often lack adequate communication regarding medication use. The first gap, underdevelopment of data reciprocity across clinical and home settings, gives rise to the second problem of poor adherence outcome. Ironically, the challenge of accessibility and usability is exacerbated by innumerable handheld device apps that are accessible, timely, and easily understood, and are increasing in number. As of October 2013, for example, 160 apps were identified as being available and focusing on an aspect of medication adherence. The sheer number of available apps makes it difficult for patients and providers alike to identify the best solution to address their unique needs. Moreover, creating a system to support sharing of data between this multitude of apps and an array of EHR systems is daunting. Given that medication management requires many participants, roles, responsibilities, and handoffs, data capture that is meaningful for patients is often lost in the chaos of medication management as care processes cross boundaries and settings of care. Thus, criteria for evaluating and encouraging medication data reciprocity are warranted.

An example of underdevelopment of data reciprocity is demonstrated in the CMS-funded Southeastern Diabetes Initiative (SEDI) program [16]. This ongoing project consists of behavioral strategies to support medication-taking that are broadly implemented through clinics, community venues, and patient home visits in four southeastern US counties in North Carolina, Mississippi, and West Virginia. The EHRs in each of the four county sites lack designated fields for documenting patient participation in medication management and self-care support interventions. This gap renders the task of measuring and collecting feedback regarding the impact of medication related care processes impossible, and key stakeholders such as community health workers, nurses, dieticians, physicians, and pharmacists lack data to support communication and provide feedback to patients regarding participation in adherence interventions.

A solution, one which obviates the need to directly link disparate health information systems containing sensitive protected health information (PHI), lies within the range of possibilities presented by Meaningful Use Stage 3. Such a solution would require functionality in the form of common discrete identifiers for EHR fields to indicate patient-selected medication management strategies and the discrete identifiers for community-based resources that were used to access, deliver or monitor the management strategy. For example, participation in the Diabetes Self-Management Program [17,18] would be documented independently by county providers in the EHR, but the patients’ enrollment and actual attendance in such programs would be in a sortable, discrete, commonly labeled data field in the EHR, regardless of county. Subsequent changes in glycated hemoglobin (HbA1c) could be easily identified and evaluated, both at the individual and aggregate levels, and would be available immediately. In this way, EHR functionality is linked with medication management outcomes across settings, from inpatient to outpatient, and across systems of care from county to county.

Improving linkages and data access will likely improve clinical care. Linkages provide an effective method to evaluate quality indicators related to medication adherence including patient-centered outcomes (PCOs) that are associated with improved medication adherence. Better integration and capture of PCOs will become increasingly important as they are integral components of a more comprehensive approach to patient care, and include objective measures of medication-related clinical outcomes (eg, blood sugar, glycated hemoglobin, blood pressure, and lipids). In addition, the patient-reported outcomes associated with medication management including knowledge, side effects, beliefs of medications are important subjective indicators of patient engagement, and progress in medication-related goals. Patient-reported outcomes (eg, cost-motivated medication non-adherence, barriers to adherence) assessed via validated surveys and questionnaires are currently in use in many clinical settings, but are inconsistently represented in available electronic formats. As a result, patient-reported outcomes are inaccessible for research and unable to be monitored for data quality. These patient-reported outcomes include common measures such as the (hospital) Consumer Assessment of Healthcare Providers and Systems (CAHPS) surveys, health literacy measures, and medication adherence measures such as the Morisky Medication Adherence Scale [19] or the Medication Discrepancy Tool [20,21]. Improved linkages and data access will therefore improve the use in clinical care of patient reported outcomes data associated with medication-taking that are collected across healthcare systems and clinical data networks.

In summary, the state of the science of HIT falls short of this definition of interoperability. Though the phases of meaningful use implementation have pushed increasing numbers of hospitals including critical access hospitals and federally qualified health centers, to invest in EHRs, these systems are challenged by weak links in the data definitions across electronic platforms and low levels of interpretability and access by both physicians and patients [22,23]. Thus, the potential beneficial impact of HIT on medication adherence has not been achieved.

Solutions will require functionality in the form of discrete identifiers that are commonly defined and consistently adopted and applied at each point of data contact. These points of contacts will range from data capture, to coding, to data transmission, and include outpatient data such as pill-taking history, medication refill rates, and patient-reported experience of side effects. Each of these data components must be accessible, timely, and easily understood by users.

### Inconsistent Data Definitions

Across almost every level from descriptive evaluation of medication fill-rate patterns and trends [24] to complex predictive modeling of the association of medication adherence with clinical outcomes [25,26], methods for analysis of medication adherence fail to meet patients’ needs and expectations. This relative lack of progress is due in part to issues surrounding the data itself. The variability in data definitions and inconsistency in terms used in practice and research prevent successful application of interventions from controlled research settings to real-world populations [27,28]. "Data definition" is the electronic specification established for keyed data entry of each data element. For example, blood pressure must be specified by type (arterial systolic, diastolic or mean), source (cuff, intra-arterial or venous), and limits on digital display (eg, two decimal places). Implications of underdeveloped data linkages are apparent when examining current methods for the evaluation of interventions to improve medication use. In addition, the definitions and terms most commonly used to evaluate medication use in research have not been conceptualized from a patient perspective [29,30]. Lastly, measurement and data capture from the many entities that contribute to management of adherence over time, including patients, caregivers, providers, communities, and health systems, are inaccessible or absent [31]. Gaps exist in the validity, accessibility, and efficiency of data sources commonly used to study adherence interventions and improve patient management of medications.

An example of inconsistent data definitions for medication adherence is the distinction between initiation, renewal, and therapeutic discontinuation. For many HIT systems, there is no mechanism in the health IT infrastructure that allows a prescriber to note that they are initiating a prescription for the first time (new to therapy) versus a refill authorization or new prescription for therapeutic continuation. While there is an existing mechanism for a prescriber to declare that they are discontinuing a medication in the 10.6 SCRIPT e-prescribing standard, for example, vendors have been slow to adopt this feature due to lack of a clear directive through meaningful use standards to date. Clinical programs such as medication reconciliation and pay for performance (eg, Medicare Star Ratings) could undergo dramatic improvements in precision through increased development, standardization, and incentive programs for adoption of order entry capabilities that have explicit data on medication initiation and discontinuation.

The underdevelopment of data linkages has major impediment for improving health care quality and patient outcomes. An outdated or incomplete medication list may give health care providers insufficient or inaccurate information to provide proper medication management of patients’ conditions. Similarly, there may be insufficient or inaccurate data to understand a patient’s non-adherence. Without incentives for medication reconciliation, this problem may continue unchecked. By not having consistent data definitions for non-adherence, it is unclear what would be "flagged" and trigger intervention from the health care system. Is non-adherence filling a prescription seven days too late or not at all? If a patient fails to fill a new prescription, does that constitute non-adherence? Having consistent data definitions would improve understanding of a patient’s ongoing medication use, which would in turn have the potential to improve symptom control and reduce morbidity and mortality.

The inconsistent data definitions and lack of harmonized data standards make tracking data relevant from a patient’s perspective difficult. These data sources include a patient’s perceptions of the medication, barriers and/or facilitators to taking the medication, and potential side effects; all factors likely to impact medication adherence. These data sources lack harmonized data definitions and data linkages across settings, resulting in poor accessibility of data from outpatient, community, and home settings to hospital and pharmacy dispensing industry settings. As a result, data are difficult to use, and when they are accessible, the data quality is poor and not amenable for use in research.

In addition to having inconsistent data definitions, there is also lack of agreement regarding where data should be stored; drug therapy problems typically do not have a place to reside within EHRs. Drug therapy problems are typically not included in the more general problem list within EHRs and other electronic systems. Many commonly understood drug therapy problems are the direct progenitor for the patient non-adherence and are critical pieces of information that are neither stored nor shared across HIT systems.

Challenges in identifying common data definitions that are meaningful to patients stem from the etymological origin of the terms used in research and practice to reflect "medication-taking". These terms were not derived from a patient perspective. Thus, terms that are most commonly used and most likely to be well defined and standardized [32], such as medication possession ratio or proportion of days covered, do not reflect aspects of medication-taking that are considered important or useful to patients. As a result, study questions and adherence interventions are less likely to be designed from a perspective that will yield meaningful information for patients.

Thus, solutions need to include terms, data definitions, and data standards that are clearly defined, standardized, and oriented from a patient-centric view of medication management in everyday life.

### Inability to Effectively use the National Drug Codes

A third major barrier to the use of HIT for medication adherence in the United States is the inability to effectively use the National Drug Code (NDC) information (US Food and Drug Administration (FDA), 2013) from the various EHR and claims datasets for adherence purposes. Though NDC codification provides a unique 10-digit drug identifier with three segments for each FDA approved medication, only the first segment is a fixed identifier from the FDA, while the second and third segments vary by company and product. As a result, although NDC codification is intended to greatly improve data quality, the 3-segment numeric indicator of the vendor, product specification (strength, dose, and formulation of the drug), and trade packages are not standardized. Thus, the stage at which the codes are implemented in the EHR system is critical [33].

One solution to address the inability to effectively use the NDCs could be improved through standardized codes across all contexts that are integrated into the EHR, much like existing International Classification of Diseases (ICD) codes.

### Capture of Medication Management Therapy

A fourth challenge with HIT in regards to medication adherence is the poor capture of medication management therapy (MTM) in current electronic data systems. MTM ensures optimum therapeutic outcomes through improved medication adherence, reduces the risk of adverse events, is developed in cooperation with licensed and practicing pharmacists and physicians, and is coordinated with any care management plan established for a targeted individual under a chronic care improvement program [34]. Specific activities of MTM include performing a comprehensive medication review, formulating a treatment plan, and providing patient education to promote adherence, among other services. Although MTM is part of Medicare Part D, data regarding provision of MTM services is not routinely captured and is not available (eg, "traceable") beyond the initiation of the checkbox at the pharmacy. Functionality "fix" to address this gap would be to link MTM data from pharmacy databases to the EHR such that actionable alerts could be routed to the physician message basket or "inbox", and summaries of educational interventions would be logged in the outpatient interactions log and tagged with "pharmacy provider" label for rapid identification. In this way HIT connectivity closes the communication loop and meets guideline measures for communication and information exchange as outlined in meaningful use stage three.

Some of the data elements that are most important to patients are not captured at all. For example, the data elements reflecting daily management of medications by patients, such as the implementation of routines and reminders to facilitate medication-taking, the monitoring of daily physiologic indicators that drive dose, such as blood sugar in diabetes or daily weight in heart failure, or the access to pharmacies with home delivery; these details of daily management significantly alter the ability of patients to manage medicines. Yet, most are not captured, or if captured are not transmitted to providers in a way that enables feedback using real-time data. The Medicare Part D Medication Therapy Management program is limited to measures of cost and resource utilization. Though one study has reported improvements in patient outcomes [35], these types of intervention outcomes for management of medicines are not consistently documented and addressed.

Thus, one solution to the poor capture of MTM in current electronic records would be to link MTM data from pharmacy databases to the EHR such that actionable alerts could be routed to the provider message basket or "inbox". These messages could summarize outpatient interactions and be tagged with "pharmacy provider" label for rapid identification.

### Role of Mobile Apps, HIT, and Medication Adherence

In addition to the challenges laid out above, consideration of how mobile medical apps will play into the mix of operability, HIT, and medication adherence are needed. The US FDA issued final guidance for developers of mobile medical apps, which are software programs that run on mobile wireless communication devices and perform the same functions as traditional medical devices. The FDA intends to focus its regulatory oversight on a subset of mobile medical apps that present a greater risk to patients if they do not work as intended. For example, an app that allows a health care professional to make a specific diagnosis by viewing a medical image from a picture archiving and communication system on a mobile phone or tablet. Another example requiring FDA regulation would be transforming a mobile platform into a regulated medical device such as an app that turns a mobile phone into an electrocardiography machine to detect abnormal heart rhythms or determine if a patient is experiencing a heart attack. However, further consideration of how mobile apps can interact with electronic medical records as well as provide more information than simply reminders is needed. For example, consider a mobile app is able to collect information from the patient regarding chemotherapy side effects they may be experiencing, then uses an evidence-based algorithm to prioritize these side effects, and integrates the prioritized list into the EHR for the provider to evaluate.

## Discussion

A system-based view of overall medication use, management, and patient adherence is needed. Improving medication use is a systems challenge, given the many entities involved in the whole process. Patients reliant on medications are tied to the prescriber’s office, the dispensing pharmacy, their home, their health plan, prescription drug plan, and pharmacy benefit management. A system-based view of medication use would be a first step towards building a model that would allow stakeholders to track a patient’s experience with medications over an entire continuum of care, and allow stakeholders to visualize how and when different interventions are (or should be) delivered to patients over time.

Until the challenges of data complexity are addressed, health care providers may be reticent to have medication use data incorporated into the EHR, unless there is clear guidance on how to respond to various lapses in medication use, especially as provided for in real-time. There may be additional medical liabilities that might occur if providers fail to respond to a prompt indicating non-adherence, which results in a negative outcome for the patient. Currently, liability for non-adherence lies primarily with the patient. Integrating these data into the EHR, as proposed, will likely shift liability to the provider and require that expectations and standards for response roles and timing be considered.

Finally, to bring a system-based perspective to bear on complex data and data integration issues, we summarize six recommendations for solutions to move HIT to the next phase of use (Textbox 1). These six recommendations are not an exhaustive list, but encapsulate the evidence to date and the opportunities that lie ahead. Importantly, these potential solutions present an opportunity for collaborative work across key stakeholders, including patients, providers, pharmacists, payers, and health technology programmers, designers, and developers. Building broad, collaborative, and multidisciplinary working groups to address these issues is the next exciting frontier in health.

**Recommendations to leverage HIT to improve medication use and adherence.**

Recommendation

1. Improve HIT interoperability by designating common discrete fields reflecting medication management.
2. Develop consistent data definitions for medication management activities and outcomes.
3. Increase use of national drug code by using these codes for prescribing and dispensing.
4. Develop data capture in EHR for MTM and allow the sharing of MTM strategies to providers across the system. In addition, having the ability to acknowledge that further clarification regarding medication if need be and allowing the sharing of this information to providers in the patients’ health care network.
5. Leverage integration of mobile apps to improve patient self-monitoring data capture and associated provider feedback when appropriate.
6. Capture the data linkages for medication adherence from the provider perspective that highlights therapeutic initiation, continuation, and discontinuation.

## Abbreviations

CMS: Centers for Medicare and Medicaid Services
EHR: electronic health record
FDA: Food and Drug Administration
HIT: health information technology
MTM: medication management therapy
NDC: National Drug Code
PCO: patient-centered outcome
